# Characterization of effects of genetic variants via genome-scale metabolic modelling

**DOI:** 10.1007/s00018-021-03844-4

**Published:** 2021-05-05

**Authors:** Hao Tong, Anika Küken, Zahra Razaghi-Moghadam, Zoran Nikoloski

**Affiliations:** 1 Bioinformatics Group, Institute of Biochemistry and Biology, University of Potsdam, Potsdam, Germany; 2 Bioinformatics and Mathematical Modeling Department, Centre for Plant Systems Biology and Biotechnology, Plovdiv, Bulgaria; 3 Systems Biology and Mathematical Modeling Group, Max Planck Institute of Molecular Plant Physiology, Potsdam, Germany

**Keywords:** Single-nucleotide polymorphisms, Metabolic models, Genome-wide association studies, Genomic selection

## Abstract

Genome-scale metabolic networks for model plants and crops in combination with approaches from the constraint-based modelling framework have been used to predict metabolic traits and design metabolic engineering strategies for their manipulation. With the advances in technologies to generate large-scale genotyping data from natural diversity panels and other populations, genome-wide association and genomic selection have emerged as statistical approaches to determine genetic variants associated with and predictive of traits. Here, we review recent advances in constraint-based approaches that integrate genetic variants in genome-scale metabolic models to characterize their effects on reaction fluxes. Since some of these approaches have been applied in organisms other than plants, we provide a critical assessment of their applicability particularly in crops. In addition, we further dissect the inferred effects of genetic variants with respect to reaction rate constants, abundances of enzymes, and concentrations of metabolites, as main determinants of reaction fluxes and relate them with their combined effects on complex traits, like growth. Through this systematic review, we also provide a roadmap for future research to increase the predictive power of statistical approaches by coupling them with mechanistic models of metabolism.

## Introduction

Advances in genotyping have provided unprecedented insights in the genetic variations among individuals of the same species. Allelic variations within a genome of the same species include the differences in the number of tandem repeats at a particular locus, segmental insertions/deletions (indels), and single-nucleotide polymorphisms (SNPs) [1]. Since SNPs represent the most abundant form of allelic variations [2], they represent the predominant factor that induces phenotypic differences among individuals. Usage of SNPs with modern machine-learning approaches have revolutionized molecular plant breeding, both with respect to applied research in prediction of traits and basic research in the mechanisms governing a trait [3–5]. Hence, characterising the effects of SNPs on agronomically relevant traits is a key problem in the interlinked fields of plant systems biology, quantitative genetics, and plant breeding.

Depending on their genomic location, SNPs have the potential to alter all steps of transcription and translation. For instance, if a SNP lies in a transcriptional regulatory element, it can alter mRNA expression; in addition, SNPs that do not lie in protein-coding regions can affect splicing, mRNA degradation, and the sequence of non-coding RNA. If a SNP that lies in a protein-coding region is synonymous, i.e. does not cause amino acid change, it can affect the translation rate and turnover of mRNA, ultimately reflected in changes of the protein abundance; finally, if the SNP is nonsynonymous (missense or nonsense), i.e. leads to amino acid change, it can modify the protein activity. Through their effects on mRNA, enzyme abundance and stability as well as enzyme activity, SNPs have direct effect on metabolic reactions catalysed by the respective enzymes.

Metabolism represents the entirety of biochemical reactions through which nutrients are imported from the environment and are transformed into the building blocks of biomass, ensuring growth, as well as other cellular components that support defence, reproduction, and, ultimately, survival [6]. A quantitative characteristic of a metabolic reaction is its rate. The rate or flux of a reaction denotes the speed at which it transforms the substrates into products [7]. The flux of a reaction depends on the abundance, $E$, of the enzyme that catalyses the reaction, its turnover number, ${k}_{\mathrm{cat}}$, representing the number of substrate molecules that each active site of the enzyme converts to product molecules per unit time, and the concentration of metabolites, $x$, acting as substrates and/or effectors (e.g. allosteric regulators, inhibitors). In the most general form, the flux of a metabolic reaction $r$ can be mathematically written as:

$$v\left({k}_{\mathrm{cat}}, {\varvec{K}},E,x\right)={k}_{\mathrm{cat}}E\eta \left({\varvec{K}},x\right)= {V}_{\mathrm{max}}\eta \left({\varvec{K}},x\right),$$

where ${\varvec{K}}$ denotes a set of parameters (e.g. Michaelis–Menten constants, ${K}_{\mathrm{m}},$ equilibrium constants, ${K}_{\mathrm{eq}}$), ${V}_{\mathrm{max}}$ is the maximal enzyme activity, and $\eta \left({\varvec{K}},x\right)$ is a function that models the effect of metabolite concentration on the flux.

**Fig. 1:**
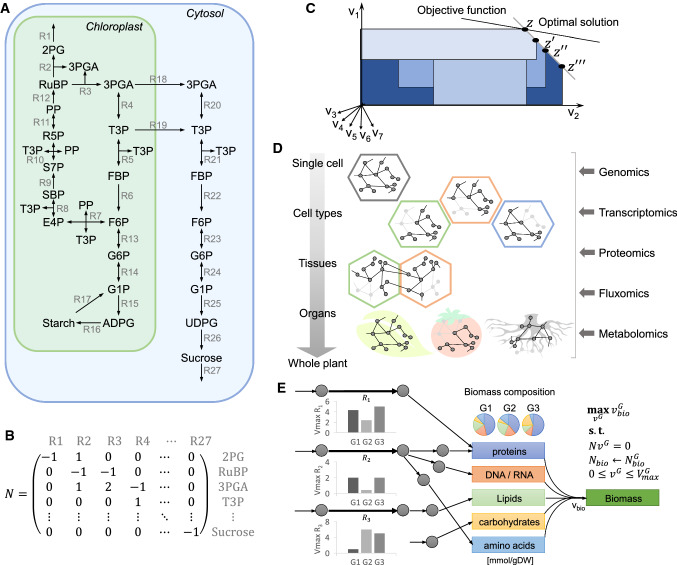
Concepts from constraint-based modelling of metabolic networks. **a** Simplified metabolic network of the Calvin–Benson cycle, starch and sucrose synthesis including two compartments (chloroplast and cytosol), 27 reactions and 24 compartment-specific metabolites. All triose-3-phosphates are lumped in a common pool denoted by T3P. **b** The concept of the stoichiometric matrix *N* on reactions R_1_ to R_4_ and R_27_ from (**a**). **c** The system of linear equations representing the metabolic model has multiple solutions, forming the solution space. Data-driven constraints can be included to reduce the solution space, each resulting in a smaller subspace. **d** Integration of data from various technologies/approaches (genomics, transcriptomics, proteomics, fluxomics, and metabolomics) allow the reconstruction of cell type-, tissue- or organ-specific metabolic networks. **e** Data on maximal reaction rates (${V}_{\mathrm{max}}$) and biomass composition for different genotypes (here G_1_, G_2_, and G_3_) can be used to further refine the predictions from metabolic networks to obtain genotype-specific flux estimates. Metabolite abbreviations: 2PG—2-phosphoglycerate, RuBP—ribulose-1,5-bisphosphate, 3PGA—3-phosphoglycerate, T3P—triose-3-phosphates, FBP—fructose-1,6-bisphosphate, F6P—fructose 6-phosphate, G6P—glucose 6-phosphate, G1P—glucose 1-phosphate, ADPG—ADP-glucose, UDPG—UDP-glucose, PP—pentose-5-phosphates, E4P—erythrose-4-phosphate, SBP—sedoheptulose-1,7-bisphosphate, S7P—sedoheptulose-7-phosphate, R5P—ribulose-5-phosphate

Metabolic reactions do not operate in isolation and jointly affect the temporal change of metabolite concentrations (Fig. 1a). A metabolic reaction can be described by the stoichiometry of its substrates and products, yielding the stoichiometric matrix, $N$, over all reactions (Fig. 1b). The change of metabolite concentrations over time can then be modelled as $\frac{\mathrm{d}x}{\mathrm{d}t}=Nv$, where $v$ gathers the fluxes of all reactions in the modelled metabolic network. Correspondingly, we can categorise the effect of SNPs on reaction fluxes into local, affecting ${k}_{\mathrm{cat}}$, and global, via transient effects of SNPs on enzyme abundance, $E$, metabolite concentrations, $x$. Given the role of reaction fluxes in shaping the main components of growth and other cellular tasks important for survival, it is paramount to determine the effects of SNPs on reaction fluxes and to further dissect them into local and global.

Reactions can be divided into extra- and intracellular based on whether or not they facilitate the exchange (i.e. import or export) of metabolites with the environment. Monitoring the change of extracellular metabolite concentrations over time can be readily used to estimate extracellular reaction fluxes [8]. However, intracellular fluxes are more difficult to quantify, and require setting up isotope labelling experiments and measurement of metabolite labelling patterns which are then fitted to a metabolic model [9, 10]. In plants, the problem is further complicated by the fact that time-resolved metabolite labelling patterns from feeding ^13^CO_2_ are required to infer intracellular reaction fluxes in photoautotrophic growth [11, 12]. Therefore, isotope labelling experiments are currently too laborious to allow estimation of fluxes in a population of individuals from a given species, rendering it infeasible to dissect the genetic architecture of fluxes in different model plants and crops following this approach.

As a result, other computational approaches have been developed to predict/estimate fluxes in the constraint-based modelling framework based on the assumption that an organism optimises a cellular task (e.g. growth) under a set of physicochemical constraints [13] (Fig. 1c). This is the essence of flux balance analysis (FBA) which provides efficient means to estimate fluxes based on constraints from measurement of extracellular fluxes and growth of microorganisms [14]. Extension of FBA has led to variants that include additional assumptions capturing efficient usage of cellular resources [15]. Interestingly, this parsimonious strategy is also often followed in application of constraint-based modelling approaches with plant metabolic networks [16, 17]. In contrast to the isotope labelling experiments above, constraint-based approaches provide a feasible means to begin to unravel the genetic determinants of reaction fluxes in plants and to use them in plant breeding.

Here, we review a collection of recent modelling approaches, which allow the dissection of the genetic basis of reaction fluxes by identifying their association with SNPs that are integrated into metabolic networks. Since these approaches can be grouped based on whether or not they rely on the principles underlying genome-wide association and genomic selection, we also describe the basic methodology underlying these machine-learning and statistical approaches. Focusing on the global effects of SNPs, we also provide a succinct review of studies that examine SNP effects on maximal enzyme activity in model plants and crops. We then offer a perspective for determining local effects of SNPs on ${k}_{\mathrm{cat}}$’s by coupling of proteomics technologies and modelling approaches in diversity panels. Finally, we point out how these modelling approaches can help address the transferability of statistical models to make predictions of traits in unseen environments by their integration into mechanistic models of metabolism.

## Constraint-based metabolic models of model plants and crops

Access to a high-quality metabolic model of an organism is key to accurate estimation of fluxes. Genome-scale metabolic models (GEMs) gather the entirety of documented metabolic reactions assembled based on annotation of the genome of an organism [18]. GEMs are further refined to include cellular compartments by considering information of protein localization and intracellular transporters. GEMs usually include a synthetic reaction, called biomass reaction, that expresses biomass as a defined ratio of macromolecules synthesised from metabolites, assembled from genome annotation and metabolomics measurements [19]. Since metabolism differs between cell types, tissues, and organs, omics data (e.g. transcriptomics, proteomics, and metabolomics) from these cellular context have been used in combination with constraint-based approaches to extract respective context-specific metabolic networks [20] (Fig. 1d).

**Fig. 2:**
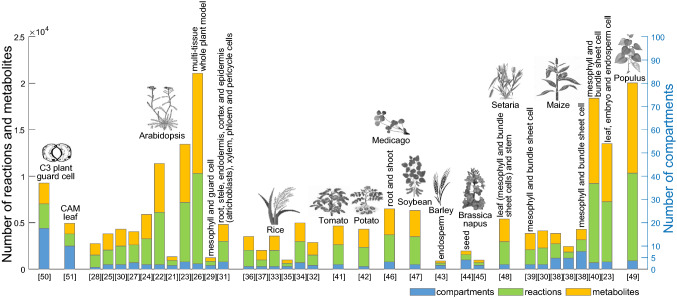
Overview of available plant metabolic network reconstructions. The existing stoichiometric models of model plants and crops are compared based on the number of compartments, metabolites and reactions included

Efforts in the last decade have resulted in the assembly of high-quality GEMs and metabolic models of central carbon metabolism for key model plants and crops, including: *Arabidopsis thaliana* (Arabidopsis) [21–31], *Oryza sativa* (rice) [32–37], *Zea mays* (maize) [23, 30, 38–40], *Solanum lycopersicum* (tomato) [41], *Solanum tuberosum* (potato) [42], *Hordeum vulgare* (barley) [43], *Brassica napus* (oilseed rape) [44, 45], *Medicago truncatula* [46], *Glycine max* (soybean) [47], *Setaria viridis* [48] and *Populus trichocarpa* [49], as well as generic models for CAM, C_3_, and C_4_ plant species [50–53] (Fig. 2). The models differ with respect to whether they only include pathways from primary metabolism (e.g. AraCore in Arabidopsis [21]) or they also consider pathways of secondary metabolism (e.g. the Arabidopsis model of Mintz-Oron et al. [27]). Further, the models include different details of representation of the underling biochemical reactions, which particularly holds for lipid metabolism [54]. They also differ with respect to the number of cellular compartments modelled and genes included (Fig. 2). The latter is particularly important if missense SNPs are to be integrated in these models following the gene–protein–reaction (GPR) rules, modelling the relation between genes, their products, and the reactions they catalyse [18]. Further, based on these GEMs, context-specific metabolic networks have already been extracted for Arabidopsis cotyledon, flower bud, open flower, root, juvenile leaf and silique [27], mesophyll and guard cells [29, 50], root cell types [31], as well as mesophyll and bundle sheath cells in maize, along with models of maize leaf, embryo, and endosperm [23, 40]. These models have been used to make predictions and further analyse genome-scale flux distributions under different growth scenarios [16].

Determining the genetic basis of the flux for a given reaction requires that it is quantified in multiple individuals (i.e. genotypes) whose metabolic networks may differ (Fig. 1e). To this end, availability of quantitative metabolomics data from different individuals allow the possibility to set up genotype-specific biomass reactions [55, 56]. In addition, measurements of extracellular fluxes across individuals as well as maximal enzyme activities, ${V}_{\mathrm{max}}$, can be used to establish genotype-specific constraints on the model’s input and output and intracellular fluxes [55]. Further, omics data from different genotypes in conjunction with context-specific model extraction approaches, mentioned above, can be used to extract more refined genotype-specific models. The resulting genotype-specific metabolic models together with constraint-based modelling approaches [14] can be used to obtain genotype-specific flux distributions, as a first step in dissecting the genetic basis of individual fluxes.

## Statistical approaches for linking SNPs with metabolic traits

Establishing a link between genetic markers (e.g. SNPs) and a trait of interest is carried out by application of machine-learning and statistical approaches. Two principal questions can be posed: (1) is the trait statistically associated with a genomic region or position? (2) Are the genetic markers predictive of the trait? (in the sense of predicting a major proportion of the variance). These questions can be used to group the statistical approaches to link genetic markers with (metabolic) traits into those that aim to conduct genetic mapping and those that devise models for genomic selection, respectively.

### Genetic mapping approaches

**Fig. 3:**
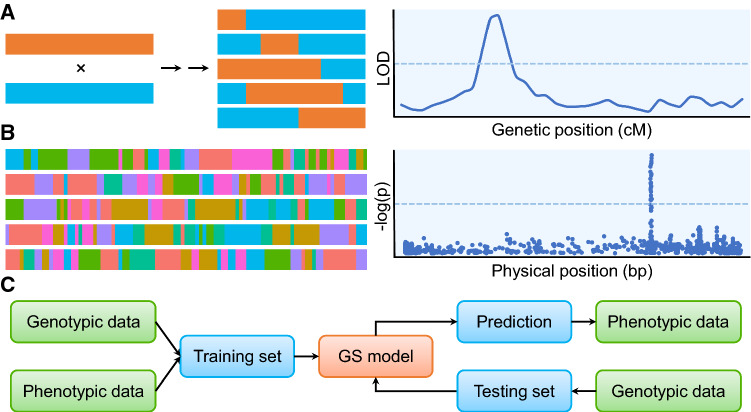
Statistical approaches for linking SNPs to (metabolic) traits. **a** Biparental mapping population based on crossing of parents that show differing values for a trait of interest together with a LOD scores for regions associated with the trait. **b** GWAS population composed of genetically diverse genotypes along with a Manhattan plot showing the *p* value of the SNPs used in mapping. **c** The process underlying genomic selection, in which genotypic and phenotypic data in a training set is used to train a statistical model for a studied trait, followed by application of the model to a testing population that is only genotype to predict respective phenotypes

Genetic mapping of a given trait can be used to determine and dissect the genetic architecture of the trait. Therefore, it provides a useful approach to improve crop breeding towards generation of better performing genotypes [57]. An essential requirement for genetic mapping is having access to a population with available genotypic data, describing the genetic variation, and phenotypic data for studied traits. Genetic mapping consists of five steps: (1) design or select a population, (2) collect the genotypic and phenotypic data, (3) conduct a screen based on statistical genetic models, (4) prioritise the significant signal for candidate genes, and (5) validate candidate genes [58]. Based on the population employed, the statistical models used to link the genotypic with phenotypic variation can drastically differ: the approach using biparental populations (e.g. F_2_ populations, backcross, and recombinant inbred lines (RILs)) is termed as quantitative trait loci (QTL) mapping (Fig. 3a), while that using natural populations (i.e. diversity panels) is at the core of genome-wide association studies (GWAS) [59] (Fig. 3b). Preprocessing of data on multiple traits based on principal component analysis can be also used to derive linear combinations of traits as latent variables, which can also be used in mapping [60].

#### QTL mapping

QTL for a studied trait denotes genomic regions that control the trait. QTL mapping relies on using low-density genetic markers, e.g. amplified fragment length polymorphism (AFLP), restriction fragment length polymorphism (RFLP), and simple sequence repeat (SSR), because the recombination blocks in biparental populations are relatively big. This approach has provided powerful means to identify loci that co-segregate with the studied trait in the employed biparental population, due to the smaller number of false positive candidates [59]. However, the resolution of QTL mapping is relatively low as it depends solely on the recombination events that occur during the process of generating the population [61]. Multi-parent populations can increase the mapping resolution [62–64], but require high-density genetic markers which can readily be obtained with modern cost-effective genotyping technologies. The statistical approaches for QTL mapping are based on the linkage map, which is the order of markers on chromosome and genetic distance between marker pairs. The most widely used QTL mapping model is composite interval mapping (CIM) model that considers the covariates to eliminate the effect of markers outside the tested interval [65] (Fig. 3a).

#### GWAS

In contrast to QTL mapping, GWAS has a relatively higher resolution, often down to a single gene level, since it relies on high-density SNPs covering the entire genome [66]. Therefore, GWAS has been the method of choice to dissect the genetic architecture of quantitative traits in animals and plants in the last decade [67–69]. The evolutionary history of diversity panels leads to accumulation of alleles that are in linkage disequilibrium, allowing to detect association between genotype and phenotype. However, the power of GWAS to detect true associations can be affected by at least five factors: (i) the mapped trait should exhibit (approximately) normal distribution, (ii) size of the population, which is related to the proportion of associations of higher effects, (iii) population structure, which leads to identification of spurious associations, (iv) allele frequency, that affects the resolution power, and (v) linkage disequilibrium, that assists in defining the significantly affected loci [59].

To address the issues of population structure and relatedness present in natural population, a mixed linear model (MLM) including kinship matrix and population structure was proposed [70], which is the most common used GWAS model in plants. The model is given by

$$y=X\beta +S\alpha +Qv+\xi +e,$$

where $y$ is a vector of phenotypic data, $X\beta$ is the intercept other than SNP effect and population structure, $S$ is a design vector for each SNP, $\alpha$ is the SNP effect, $Q$ is the population structure matrix, $v$ is the population structure effect, $\xi \sim MVN(0,K{\sigma }_{u}^{2})$ is the polygenic effect, and $e$ is the residual error. The population structure can be revealed by approaches based on principal component analysis [71]. The polygenic effect and residual error are treated as random effects, while the others are fixed effects. Therefore, the variance of $y$ is

$$V=K{\sigma }_{u}^{2}+I{\sigma }_{e}^{2},$$

where $K$ is the kinship matrix, $I$ is the identity matrix, ${\sigma }_{u}^{2}$ and ${\sigma }_{e}^{2}$ are the variance component of polygenic effect and residual errors, respectively. These variance components are estimated by restricted maximum likelihood (REML) approach. The best linear unbiased estimation (BLUE) of fixed effects and best linear unbiased prediction (BLUP) of random effects are then calculated. The test of significance is performed by the *F* test or likelihood ratio test between the model without consideration of the SNP effect and the model that includes the tested SNP (Fig. 3b). The test of significance is carried out in a single locus analysis, so a multiple test correction must also be performed.

However, application of the MLM approach is computationally challenging with the increase in the number of samples and SNPs that are required to improve the resolution and power of the genetic mapping. Several efficient GWAS algorithms have been devised to handle the population structure and kinship by employing elegant matrix transformations (e.g. the efficient mixed model association (EMMA) [72], genome-wide EMMA (GEMMA) [73], and factored spectrally transformed linear mixed model (FaST-LMM) [74]). In contrast to the above methods, other algorithms estimate the polygenic effect only once, and keep it constant for every tested SNP (e.g. population parameters previously determined (P3D) [75]). In addition, to avoid control the population stratification via kinship and population structure matrix, the multi-locus mixed model (MLMM) has also been used in GWAS [76].

From this brief review of computational approaches for genetic mapping based on GWAS, it is evident that they are all based on statistical approaches of association and do not consider mechanistic insights and constraints. Several pressing questions arise: can the coupling of the basic principles of GWAS with mechanistic models of metabolism help in detecting causal SNPs with local effects on reaction fluxes? If so, could this be done with smaller population sizes, without reducing the power of the detected associations? These questions will be addressed in “Approaches based on GWAS”.

### Genomic selection

Genomic selection (GS) is considered the most promising breeding method to speed up the development and release of improved genotypes [77]. It is based on a model to arrive at genomic estimated breeding value (GEBV) based on usage of genome-wide markers with various machine learning [78]. More specifically, GS uses machine learning to integrate phenotypic data of a given trait with molecular markers in a statistical model for a training population. The model is then employed to predict traits values of genotypes in a testing population, which have been genotyped but not phenotyped [79] (Fig. 3c). The predictions for unseen genotypes can be used for selection without any further phenotyping. Therefore, an increase in GS accuracy for agronomically important traits can accelerate genetic gain by shortening the breeding cycles [77].

In contrast to GWAS, GS forgoes statistical testing for the effect of SNPs, as the goal is to devise a model of high predictive power. Nevertheless, like GWAS, the accuracy of GS is affected by several factors, including: (i) the sample size, (ii) genetic relationship within and between the training and testing population, (iii) marker density, (iv) heritability of the trait, (v) linkage disequilibrium between markers and quantitative trait loci controlling the trait of interest, and (vi) non-additive genetic effects (e.g. epistasis) [80, 81]. It has been observed that increases in the sample size, but also changes in the structure of the training set have a strong effect on the prediction accuracy of GS [82]. Further, increases in accuracy of GS have been found to plateau after certain level of marker density [83]. GS models that take into consideration multi-environment data allow for sharing information across environments and usually lead to increase in accuracy in comparison to models derived from single-environment data [84]. However, the generation of such data takes considerable resources, so the question remains if the performance of single-environment models can be improved by modifying the modelling strategy. Finally, several studies have pointed out that epistasis is an important contributor to the long-term response to selection [85, 86]. However, while consideration of two-locus epistatic effects has led to improvements in GS accuracy [87], general consideration of epistasis in GS models remains computationally challenging and deserves further method development.

Based on the machine-learning/statistical techniques employed, GS approaches can be roughly divided into those relying on regression, classification, and deep learning techniques [5]. Ridge regression best linear unbiased prediction (rrBLUP) is one of the most common used GS models in plants [78]; it is a mixed-effect linear model, given by

$$y=Xb+Zu+e,$$

where* y* is a vector of phenotype,* X* is the fixed effect design matrix, $b$ is the fixed effect, $Z$ is a matrix of genetic markers, $u$ is the marker effect as random effect and $e$ is the residual error. The variance of $y$ is

$$V=Z{Z}^{T}{\sigma }_{u}^{2}+I{\sigma }_{e}^{2},$$

where ${\sigma }_{u}^{2}$ is the marker effect variance and ${\sigma }_{e}^{2}$ is the residual error variance. Since the number of markers is considerably larger than the number of observations (i.e. genotypes), regularisation techniques are usually used to estimate the model parameters. In comparison to ridge regression, the parameter $\lambda$ of the $l$_2_ norm is equivalent to $\lambda ={\sigma }_{e}^{2}/{\sigma }_{u}^{2}$ and penalises the ratio between the two random effect variance components. According to the mixed model theory, the value of GEBV can be solved and used to predict the phenotypic value in testing population.

This approach can shrink all effects toward zero equally across markers, under the assumption that all markers have a common variance. Other approaches, like genomic best linear unbiased prediction (GBLUP), estimate the kinship matrix from genomic markers to represent the pedigree information, then estimate GEBV in a mixed linear model that is equivalent to the rrBLUP model [88]. The GEBV can also be obtained from Bayesian statistics [78]. To compare the model performance in GS, one usually uses the prediction accuracy. It is determined by k-fold cross-validations, whereby one fold is treated as testing population and other folds as training population. The prediction accuracy is the correlation coefficient between the predicted phenotypic value and measured phenotypic value in the testing population.

Like the GWAS approaches outlined above, GS is based on machine-learning algorithms and the transferability of the resulting models to unseen scenarios, i.e. different population, different environments, and the combination of the two, remains one of the biggest challenges in the application of GS. Therefore, it is of interest to investigate if the prediction accuracy of GS for metabolic traits can be improved if the mentioned approaches are coupled with mechanistic models of metabolism, discussed in “Approach based on genomic selection”.

## Application of genetic mapping approaches for maximal enzyme activity in plants

Determining the genetic architecture of metabolism entails genetic mapping of metabolic traits, including: metabolite levels (relative and absolute content, concentrations), protein abundances and activities, and reaction fluxes. There are plethora of studies that use GWAS and QTL mapping approaches in diverse plants and crops based on measurement of metabolite levels and protein abundances [89, 90]. However, these studies rely on relative quantification of these traits, rendering it difficult to interpret the findings in terms of effects on reaction fluxes. A reaction flux depends linearly on the maximal activity, ${V}_{\mathrm{max}}$, of the respective enzyme, and is fully determined by it when the enzyme is substrate-saturated [91]. Thus, it may be expected that the results of genetic mapping of ${V}_{\mathrm{max}}$ would coincide with those of the corresponding reaction fluxes. However, due to the interconnectedness of gene regulatory and protein–protein interaction networks that affect metabolism, QTL or associated SNPs can be found not only in *cis* position (i.e. on the same chromosome and proximal) to the location of the corresponding structural genes (coding of structural proteins, rather than regulatory proteins), but also in *trans* position (i.e. on a different chromosome), denoting regulatory QTL.

To this end, all the statistical approaches for genetic mapping mentioned above can be readily used to determine the genetic architecture of ${V}_{\mathrm{max}}$ of different enzymes as well as reaction fluxes, if these are measured in an investigated population. For instance, the only study to date that has performed QTL mapping of reaction fluxes uses flux estimations from a small model of *Saccharomyces cerevisiae* (yeast) central carbon metabolism [92] based on bounds of measured extracellular fluxes and profiling of dry weight in 125 F_2_-segregants (genotyped by 3727 SNPs) from a cross of two yeast strains [93]. These approaches identified four flux QTL and two gene variants that contribute to the explanation of the variations in the flux distributions in the population.

Since intracellular fluxes are more challenging to quantify (see “Introduction”), majority of QTL mapping studies in plants have focused on dissecting the genetic basis of maximal enzyme activities. However, genome-wide profiling of maximal enzyme activities is currently not feasible, due to the limitations of the assays used [94]. As a result, these studies usually involve a handful to two dozens of enzymes, mostly covering key pathways in primary metabolism in maize, Arabidopsis, and tomato. For instance, Causse et al., Prioul et al., Thevenot et al., and Pelleschi et al. [95–98] measured the maximal enzyme activities of four enzymes, sucrose-phosphate-synthase, sucrose-synthase, sucrose-invertase, and ADP-glucose pyrophosphorylase, covering key steps in carbohydrate metabolism in sources (i.e. leaves) and/or sinks (i.e. grains) in maize RIL populations. Colocation of QTL for maximal enzyme activity and structural gene were found for sucrose-phosphate-synthase and the invertase. Limami et al. [99] measured the activity of enzymes from nitrogen metabolism, including: glutamine synthase, NAD(H)-glutamate dehydrogenase, the ferredoxin-dependent as well as the NAD(H)-dependent glutamate synthase, and phosphoenolpyruvate carboxylase in a population of 140 maize RILs, and identified QTL for glutamine synthase in the early and late stages of germination. An intermated RIL maize population was used to map QTL for the activity of ten enzymes, six from carbon and four from nitrogen metabolism [100]. All identified QTL for enzyme activities in this study were in *trans* to the respective structural genes, except for single *cis*-QTL for nitrate reductase, glutamate dehydrogenase, and shikimate dehydrogenase.

In addition, Mitchell-Olds and Pedersen [101] performed QTL mapping of maximal activity for ten enzymes (i.e. six glycolytic enzymes, glucose-6-phosphate dehydrogenase, fructose bisphosphatase, phosphoglucose isomerase, phosphoglucomutase, glucose-6-phosphatase, and hexokinase, as well as four enzymes putatively involved in defence pathways, peroxidase, shikimic dehydrogenase, myrosinase, and chitinase) in an Arabidopsis RIL population. In another Arabidopsis RIL population, Sergeeva et al. [102, 103] mapped the activity of phosphoglucomutase and sucrose-invertase. The same population was later used to dissect the genetic architecture for the maximal activity of 15 enzymes [104]; QTL were detected for 10 of the 15 enzyme activities, which exhibited higher heritability, and involved respective structural genes as well as other genes with *cis*- and *trans*-acting control. A tomato introgression population, generated by introgressing segments of the genome of the wild relative *Solanum pennellii* into the modern tomato cultivar *Solanum lycopersicum*, was used to investigate QTL for the maximal enzyme activities of 28 enzymes from central carbon metabolism [105]. To this end, measurements were conducted in the pericarp tissue of ripe tomato fruits from two field trial experiments. The identified QTL support the observations from Arabidopsis that maximal enzyme activity is under the control of *trans*-acting genes.

The only GWAS with maximal enzyme activities as a trait was carried out in an Arabidopsis diversity panel composed of 349 accessions. To this end, associated SNPs for 24 maximal enzyme activities in central metabolism were detected [106]. The study identified *cis*-QTL of moderate effects for maximal enzyme activity of five enzymes, including UDP-glucose pyrophosphorylase, ADP-glucose pyrophosphorylase, fumarase, and phosphoglucose isomerase. The remaining QTL were *trans*-acting of smaller effects than the *cis*-acting, and were found in genomic regions that include components involved in transcriptional and post-translational modifications.

Genetic mapping of maximal enzyme activities in different plant species demonstrates that genetic variants in both regulatory and structural genes can affect this trait of different enzymes in central metabolism. Therefore, consideration of missense SNPs may only identify a small fraction of the phenotypic variance in this trait. The latter implies that the integration of SNPs into mechanistic models should consider the action of *trans*-acting genes for accurate predictions of their effects on metabolic traits.

## Integration of SNPs in genome-scale metabolic models

The approaches that integrate SNPs into a metabolic network can be grouped based on two criteria: (i) if they investigate the positioning of SNPs in metabolic network, using the GPR rules and (ii) if they characterize the effect of a SNP on reaction fluxes. With respect to the second criterion, one can further subdivide these approaches based on whether they rely on principles of GWAS or genomic selection, as principal statistical approaches for linking SNPs with traits.

### Approaches based on the metabolic network structure

**Fig. 4:**
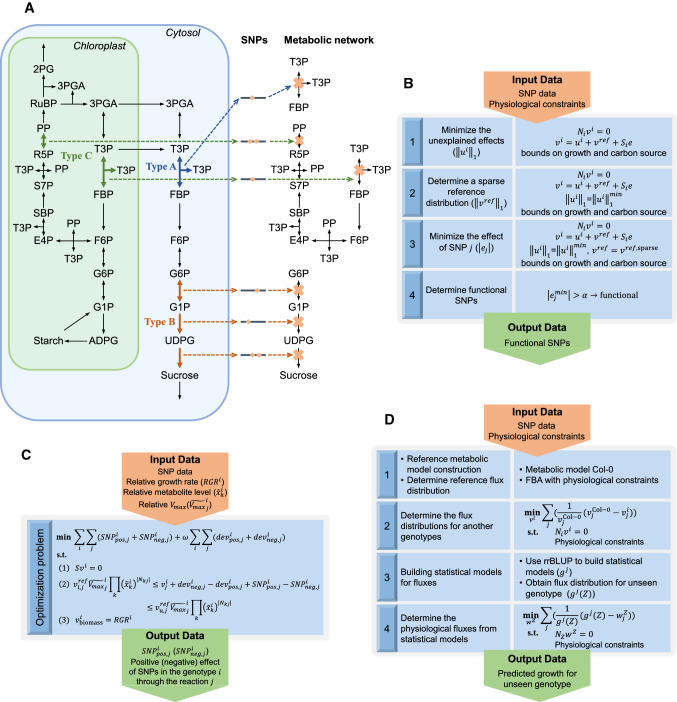
Approaches that integrate SNPs into metabolic models. **a** Examples of different types of co-sets on the metabolic network of Fig. 1a are presented by different coloured arrows. Orange diamonds show the SNPs in the gene coding for the proteins that catalyse reactions in the co-sets. The causal SNPs affect the reactions, marked by an orange x symbol for knock-out, and result in the inability of the network to produce particular products. **b** The SNP effects in [113] are predicted through three optimization steps: (1) minimising the unexplained effects, (2) finding sparse reference flux distribution, and (3) minimising the flux effect of each SNP. In the fourth step, SNPs with the minimum effects larger than the threshold of $\alpha$ are considered as functional. **c** The positive or negative effect of SNPs are captured in SNPeffect [49] by an optimization problem, in which mass-action kinetics is assumed and relative growth rate, relative metabolite level and relative ${V}_{\mathrm{max}}$ are given. **d** Four steps presented in netGS [56] allows for prediction of growth in unseen genotypes: (1) the construction of reference metabolic model and the prediction of reference flux distribution, (2) prediction of flux distributions in other genotypes by finding the closest flux distributions to the reference one, which are compatible to physiological constraints, (3) building statistical models for fluxes based on SNPs, and (4) prediction of physiological flux distributions from statistical models by finding the closest steady-state flux distribution to that obtained from the statistical models

A first approach to investigate the role of SNPs in metabolic networks is to characterize their position in the metabolic network. Due to the possibility that a metabolic reaction is catalysed by isoenzymes and protein complexes, as well as due to the promiscuity of some enzymes, whereby they can catalyse multiple reactions [107], the product of one gene can affect the flux through multiple reactions [108]. As a result, the effects of a nonsynonymous SNP on such a gene can be readily determined by investigating its position in the metabolic network. Jamshidi and Palsson indicated that the effect of SNPs that reside in genes whose products catalyse reactions that form co-sets can be readily obtained [109]. A co-set is a maximal set of reactions whose fluxes are perfectly correlated across any steady-state that the network can support [110], and coincide with fully coupled reactions from flux coupling analysis [111]. We note that a co-set can be composed of a single reaction if that reaction is not fully coupled to any other in the network. As a result, SNPs in the same co-set are expected to have similar effects. A co-set can consist of a single reaction, reactions on a linear chain, or subnetworks of more intricate structure which may also be disconnected, denoted as co-sets of types A, B, and C, respectively (Fig. 4a). While this approach is useful in providing a partitioning of SNPs based on their participation in specific subsets of reactions, it does not provide a quantification of the effect of SNPs on reaction fluxes. Interestingly, to date, there has been no characterisation of the effect of SNPs with respect to other types of dependencies between steady-state reaction fluxes [112].

### Approaches based on GWAS

One of the critical factors that determine the power of GWAS is the population size. Integration of SNPs in a metabolic network can facilitate characterisation of their effect on fluxes even with a very small population [113]. Here, we review two approaches, one based on structural sensitivity analysis and the other based on incorporation of metabolomics datasets under the assumptions about enzyme kinetics.

### Structural sensitivity analysis and GWAS

This constraint-based approach is based on structural sensitivity analysis [114], whereby the effect of a SNP in a gene is the same for every reaction that the gene product catalyses. Based on structural sensitivity analysis, the propagation of the SNP effect to the rest of the fluxes in the network can be determined. To this end, the problem is cast as a least-square adjustment of steady-state fluxes whose solution results in a sensitivity matrix, ${S}_{i}$, for the genotype $i$. The following restrictions and assumptions apply: (i) only nonsynonymous SNPs in the genes included in the metabolic network are considered, (ii) the considered SNPs are allowed to only decrease reaction fluxes (as deleterious effects of mutations are more likely), and (iii) the effect of a SNP is the same in all analysed genotypes.

With these assumptions, the approach is based on representing the genotype-specific flux distribution, ${v}^{i}$, in terms of a reference distribution, ${v}^{\mathrm{ref}}$, and deviations from it; the deviations can either be explained by the nonsynonymous SNPs, ${S}_{i}e$, or are unexplained by them, ${u}^{i}$, i.e.

$${v}^{i}={u}^{i}+{v}^{\mathrm{ref}}+{S}_{i}e.$$

In this sense, ${u}^{i}$ can be seen as a residual error that cannot be explained by the sensitivity matrix (via the effect of $e$) and the reference flux distribution. The flux distributions ${v}^{i}$ are determined for all genotypes jointly by enforcing steady-state in each, i.e. ${N}_{i}{v}^{i}=0$, using experimentally determined, genotype-specific exchange rates for a subset of metabolites. The simultaneous solving of the steady-state equations is needed due to the relation between flux distributions of different genotypes via the nonsynonymous SNP effects, given by $e$, $e\le 0$. The four steps include: (1) finding a sparse solution for the unexplained effects (via minimization of the first norm); (2) determining a sparse reference distribution (that specifies reaction fluxes in absence of SNPs); (3) minimising the effect, ${e}_{j}$, of SNP $j$ under the constraints of the spare solutions found in first two steps. This is needed due to the variability of ${e}_{j}$ in the feasible space, and helps with the interpretation of the SNP effects; and (4) only SNPs whose minimum effects are larger than an arbitrarily selected threshold $\alpha$ are considered to have functional effects (Fig. 4b). This algorithm was tested with 18 strains of the *Mycobacterium tuberculosis* complex with 556 nonsynonymous SNPs, and 88 SNPs were classified as functional with the used threshold value.

The approach can be viewed as a multi-locus GWAS [76], but does not provide statistics for associations, as it relies on the predictions from the integration of SNPs in the metabolic network. The findings from this approach depend on: (i) the number of nonsynonymous SNPs in the genotyping data, which would lead to different sensitivity matrices if the set of SNPs is altered, (ii) the number of genotypes used, as the number of variables grows linearly, leading to numerical issues with models of larger sizes, (iii) the order in which the factors, ${u}^{i}, {v}^{\mathrm{ref}},$ and $e$, of the genotype-specific flux distribution are estimated, (iv) the norm used to arrive at a sparse solution. Further, it is challenging to validate the predictions for the reference flux distribution, as it is a concept that is not tied with a particular genotype. Moreover, the SNPs are modelled as present/absent, and no distinction can be made between homozygous and heterozygote genotypes for a gene of interest. Therefore, refinements of this approach are needed to apply it in plant and crop breeding.

### SNPeffect

Like the constraint-based approach above, SNPeffect aims to determine whether a SNP is functional or not by characterising its effect on reaction fluxes [49] (Fig. 4c). The flux of a reaction $j$ in genotype $i$ is assumed to follow mass-action-like kinetics while considering enzyme action [115]:

$${v}_{j}^{i}\left({k}_{\mathrm{cat}}, {\varvec{K}},E,x\right)={{k}_{\mathrm{cat}}}_{j}^{i}{E}_{j}^{i}\eta \left({\varvec{K}},x\right)= {{V}_{\mathrm{max}}}_{j}^{i}\prod_{k}{\left({x}_{k}^{i}\right)}^{|{N}_{kj}|}.$$

As a result, the flux ${v}_{j}^{i}$ can be expressed relative to a reference flux distribution as:

$${v}_{j}^{i}={v}_{j}^{\mathrm{ref}}\frac{{{V}_{\mathrm{max}}}_{j}^{i}}{{{V}_{\mathrm{max}}}_{j}^{\mathrm{ref}}}\prod_{k}{\left(\frac{{x}_{k}^{i}}{{x}_{k}^{\mathrm{ref}}}\right)}^{|{N}_{kj}|}.$$

With measurements of available relative changes in maximal enzyme activities and metabolite levels with respect to a reference genotype, one can obtain lower and upper bounds. Deviation of the steady-state flux is then attributed to (positive/negative) additive effects of SNPs and saturation effects of the enzyme. There are three assumptions on which SNPeffect is based: (i) a SNP is assumed to have consistent effect across all genotypes, i.e. it either increases or decreases reaction fluxes, (ii) the effect of a SNP is allowed to vary across genotypes, (iii) only nonsynonymous SNPs in genes included in the metabolic network are considered. Here, the effect of a SNP are simultaneously determined over all genotypes, by including constraints of steady-state and relative growth rate with respect to the reference genotype. Implementation of the approach clearly requires setting up a reference flux distribution or specifying lower and upper bounds, ${v}_{l, j}^{\mathrm{ref}}$ and ${v}_{u, j}^{\mathrm{ref}},$ for the fluxes in the reference genotype, resulting in the following constraint:

$${v}_{l, j}^{\mathrm{ref}}{\stackrel{\sim }{{V}_{\mathrm{max}}}}_{j}^{i}\prod_{k}{\left({\tilde{x }}_{k}^{i}\right)}^{|{N}_{kj}|}\le {v}_{j}^{i}+{\mathrm{dev}}_{\mathrm{neg},j}^{i}-{\mathrm{dev}}_{\mathrm{pos},j}^{i}+{\mathrm{SNP}}_{\mathrm{pos},j}^{i}-{\mathrm{SNP}}_{\mathrm{neg},j}^{i}\le {v}_{u, j}^{\mathrm{ref}}{\stackrel{\sim }{{V}_{\mathrm{max}}}}_{j}^{i}\prod_{k}{\left({\tilde{x }}_{k}^{i}\right)}^{|{N}_{kj}|},$$

where ${\stackrel{\sim }{{V}_{\mathrm{max}}}}_{j}^{i}$ and ${\tilde{x }}_{k}^{i}$ are the relative maximal enzyme activity and relative metabolite content in genotype $i$ with respect to the reference genotype, ${\mathrm{dev}}_{\mathrm{neg},j}^{i}$ and ${\mathrm{dev}}_{\mathrm{pos},j}^{i}$ denote deviations from the assumed enzyme kinetic and ${\mathrm{SNP}}_{\mathrm{pos},j}^{i}$ and ${\mathrm{SNP}}_{\mathrm{neg},j}^{i}$ are linear combinations of SNPs denoting their negative and positive, additive effects, respectively. In the actual implementation, these constraints are simplified by assuming that ${\stackrel{\sim }{{V}_{\mathrm{max}}}}_{j}^{i}=1.$

Like in the structural sensitivity approach, above, SNPeffect can be regarded as a multi-locus GWAS in which the SNPs as present/absent, i.e. without making distinctions between different alleles. Its performance depends on: (i) the optimization function used, which in the existing implementation minimises the effects of the deviations from steady-state flux distribution that respect constraints from relative enzyme activities and metabolite levels, (ii) the reference flux distribution, determined by parsimonious FBA [15], and (iii) the number of metabolites and enzyme activities for which lower and upper bounds appearing in the expression above can be determined. In addition, SNPeffect inherits the factors that make its application challenging at a genome-scale level due to the sheer number of SNPs that can be considered. The approach was tested with models of Arabidopsis and *Populus trichocarpa* (poplar) [49], and identified functional SNPs in purine and amino acid biosynthesis pathways as well as lignin biosynthesis, respectively.

### Approach based on genomic selection

Availability of flux distributions from a population of genotypes whose size is preclusive to conduct GWAS can still be used in GS for reaction fluxes. Tong et al. [56] developed an extension to GS, called netGS, based on integration of the machine-learning models of GS in a metabolic network. netGS relies on training a machine-learning model for steady-state fluxes obtained from genotype-specific metabolic models in particular conditions. The genotype-specific models are obtained by modifying the biomass function and applying constraints with respect to the growth relative to a reference phenotype. netGS is a four-step approach: (1) a model of a reference genotype is developed and is used to obtain a reference flux distribution following constraint-based approaches, like FBA [14]; (2) a flux distribution for another genotype is obtained by assuming that the difference to the reference is minimised, while ensuring that the ratio of predicted growth rates for the two accessions matches the ratio of measured fresh weights. This steps quantifies the flux of every reaction in the investigated genotypes; (3) each reaction flux is used as a trait for GS statistical modelling (implemented as rrBLUP), resulting a model with a specific predictability; (4) since the statistical models for each flux do not result in a steady-state flux distribution when applied to an unseen genotype, netGS next finds a flux distribution compatible with biochemical constraints given the flux predictions obtained from the statistical models based on the genomic data for the unseen genotype (Fig. 4d). In such a way, netGS allows prediction of growth, via the respective biomass reaction included in the model. This constraint-based approach has also been extended to consider predictions across environments. This extension is based on the assumption that the ratio between exchange fluxes for the reference genotype in two different environments is maintained across genotypes. With this additional constraint, the developed models in one environment can be used in another.

The statistical models that are devised in the third step of netGS inherits the shortcomings of GS models. However, through forcing these models to jointly respect physicochemical constraints, netGS aims to improve the model performance for unseen genotypes and in scenarios when there are large differences between training and testing populations. The imposing of these constraints can be regarded as adjusting for epistatic interactions between SNPs, which are otherwise difficult to integrate in a statistical framework due to the large number of SNPs considered. In contrast to the approaches above, netGS is not limited to investigating only nonsynonymous SNPs, but can also consider SNPs which lie in non-coding regions of the genome—which boosts the usage of genomic data. netGS was tested with 67 Arabidopsis accessions for which genotype- and condition-specific biomass reactions were developed based on measurements. The results showed that, in comparison to classical GS, it improves the prediction accuracy of growth within and across nitrogen environments by 32.6% and 51.1%, respectively, as well as from optimal nitrogen to low carbon environment by 50.4%. The approach can readily be applied to any plant species for which metabolic models of high-quality exist and can be coupled with constraints from phenotypic data of specific genotypes.

## Roadmap for future research

The brief review of the approaches for linking SNPs with metabolic and complex traits highlighted the division of two sets of approaches rooted in different methodologies. On one side, approaches for QTL mapping, GWAS, and genomic selection are solely based on statistics; moreover, genomic selection can be regarded as a black box, machine-learning approach that does not provide mechanistic insights or candidates for further testing. On the other hand, constraint-based approaches are applicable with large-scale models of metabolism and allow to establish a link between fine-grained metabolic processes and complex traits, such as biomass accumulation and growth.

Our systematic review indicated the possibility for merging the two complementary types of approaches to overcome their principle drawbacks, namely, the need for large populations, in the case of quantitative genetics approaches, and the need for depicting phenotypic diversity in a population of genotypes, in the case of the constraint-based modelling framework. While these approaches seem to have a great potential, demonstrating their added value necessitates addressing the following issues: first, studies should be planned to compare and contrast the findings between the purely statistical approaches and those based on consideration of SNPs in metabolic models. The existing studies have not performed this comparison due to the small sizes of the populations employed. Such comparative studies would require development of approaches for extraction of genotype-specific metabolic models for which no pipelines are yet freely available. Second, as shown on Fig. 2, there exist different metabolic models for the same plant species; these models different with respect to size, details, and modelled metabolic functionalities. Thus, it will also be important to investigate the effect of the model used for integration of genotypic data. Third, the consideration of SNP effect in constraint-based modelling can potentially introduce a lot of variables; thus, it is necessary to investigate how the preselection of SNPs may affect the findings from these approaches. In addition, since constraint-based approaches are marked with alternative solutions, one would have to design procedures to explore and/or further reduce the space of alternative solutions in a meaningful way.

The prospects for coupling mechanistic and statistical modelling approaches offer several new research avenues. First, one can aim to determine the statistical significance of a SNP effect obtained from constraint-based approaches. This can be accomplished by usage of permutation tests along with the aforementioned exploration of the space of alternative solutions. As a result, one would not need to rely on arbitrarily set threshold values to classify SNPs as functionally significant. Second, similar to netGS, one can use other types of machine-learning approaches for genomic selection to partition the reactions into active/inactive or into those carrying large or small fluxes, opening the possibility for other modelling directions. Third, with the availability of algorithmic procedures for estimation of turnover numbers of enzymes in a given genotype (e.g. *A. thaliana* Col-0 [116]), one can also aim to obtain such estimates in different genotypes, opening the possibility for using genetic mapping approaches and genomic selection. The resulting statistical models can, in turn, be employed to better constraint genotype-specific models using computational approaches, such as FBA with molecular crowding [117], MOMENT [118], or GECKO [119], or by incorporating macromolecular expression (so-called ME-models) [120] that are, however, still only applied to microbes.

We envision that these milestones can be achieved in the next 5–10 years of research in metabolic modelling of crops. Altogether, such prospects for a synergistic combination of machine-learning and metabolic models will pave the way for mechanistic modelling of complex traits in populations that involve both inbred and hybrid genotypes.
